# Ion-Modified Starch Film Enables Rapid Detection of Spoiled Fruit Juices

**DOI:** 10.3390/ijms232314732

**Published:** 2022-11-25

**Authors:** Shijiao Qin, Yujia Wu, Hao Tian, Yun Liu, Huan Kan, Defa Hou, Xu Lin, Yunwu Zheng, Zhifeng Zheng, Can Liu

**Affiliations:** 1 National Joint Engineering Research Center for Highly Efficient Utilization Technology of Forestry Resources, Southwest Forestry University, Kunming 650224, China; 2 Agro-Products Processing Research Institute, Yunnan Academy of Agricultural Sciences, Kunming 650224, China; 3 Xiamen Key Laboratory for High-Valued Conversion Technology of Agricultural Biomass, College of Energy, Xiamen University, Xiamen 361102, China

**Keywords:** ion modified, starch film, drink spoilage, pH response, rapid determination

## Abstract

Juice, as a liquid foodstuff, is subject to spoilage and damage due to complications during transport and storage. The appearance of intact outer packaging often makes spoilage and damage difficult to detect. Therefore, it of particular importance to develop a fast, real-time material to evaluate liquid foodstuffs. In this paper, starch films with pH response characteristics are successfully prepared by inorganic ion modification by utilizing whole starch and amylopectin as raw materials. The mechanical properties, stability properties, hydrophilic properties and pH electrical signal response indices of the films are analyzed and measured. The films exhibit good electrical conductivity values with 1.0 mL of ion addition (10 mmol/L), causing the composite film to respond sensitively to solutions with varying pH values. In the test of spoiled orange juice, the full-component corn starch (CS) film has more sensitive resistance and current responses, which is more conducive for applications in the quality monitoring of juice. The results indicate that modified starch films can potentially be applied in the real-time monitoring of the safety of liquid foodstuffs.

## 1. Introduction

Food safety has become one of the most important issues in our daily lives. Food safety threatens human health and causes turbulence in the market economy and the stability of society; thus, food safety has a mutually balanced relationship with society and human beings [1,2,3,4,5]. Existing food safety problems are mainly caused by the following issues: heavy metal contamination in the environment [6], pesticide and veterinary drug residues [7], bacteria and biotoxins [8], additive abuse [9], and incorrect operations during production and storage. Several techniques have been developed for rapid food detection, including immunoassays [10], enzyme inhibition [11,12], colorimetric analysis [13,14], and biological techniques [15]. Although the above techniques have many advantages, they have relatively complex operations and high detection costs; the accuracy of the results is susceptible to environmental influences, leading to the deviation of the test results and making it difficult to achieve simple and accurate real-time detection. In contrast, laboratory methods for food safety detection are mainly realized by near-infrared spectroscopy [16], Raman spectroscopy [17,18], and high-performance liquid chromatography [19,20]. Most laboratory techniques require skilled technicians, and have shortcomings such as expensive instruments and long detection times, which are not conducive to rapid, simple, and real-time food detection. Therefore, it is of particular significance to develop rapid and sensitive food detection methods and materials.

Food products are damaged and spoiled during transport and storage due to complications. As a liquid food, fruit juice easily deteriorates because of technical packaging defects. Additionally, the appearance of intact outer packaging often makes damage and spoilage difficult to detect. Thus, the development of rapid, real-time materials for liquid food detection is of great significance. The deterioration of liquid foods essentially affects their pH values; therefore, a range of detection materials and techniques can be developed to pick up this particular signal. Zhang’s research group has reported an intelligent indicator label that can detect the degree of food decay [21]. Due to the pH sensitivity of the indicator label, it can be applied to detect the pH levels of liquid food; furthermore, indicator labels can detect the biogenic amines produced by rotten food in real time. Hu et al. [22] have demonstrated a pH-responsive smart food packaging antibacterial film, where the pH-responsive release behavior of the antibacterial film extends the shelf life of the pork. The above literature proves the feasibility of using pH as the main signal for food quality detection. A new research direction involves visually observing the degree of deterioration and the electrochemical parameters in testing techniques for food safety. Deng et al. [23] have proposed a graphene-modified acetylene black paste electrode for detecting bisphenol A (BPA) concentrations in foodstuffs; this electrode has a wide detection range and high sensitivity. Hao et al. [24] have designed and developed a simple portable electrochemical immunosensor with high sensitivity, stable performance, convenient storage and simple operation for the rapid on-site detection of a mycotoxin- zearalenone (ZEN) in feed and agri-food. Kundan et al. [25] have developed an ultrasensitive optofluidic surface-enhanced Raman scattering (SERS) sensor using photonic crystal-enhanced plasma capsules; the researchers have achieved the detection of the food additive rhodamine 6G and the responses to trace amounts of chlorobenzene compounds in drinking water. Smart electrical signal-responsive materials have a wide range of applications, and they have shown great promise in food safety detection applications. To date, the increasing demand for fruit and vegetable beverages has made food safety in this field extremely important. Therefore, it is urgent to develop a simple, rapid, highly sensitive and real-time method for evaluating fruit and vegetable beverages.

**Figure 1:**
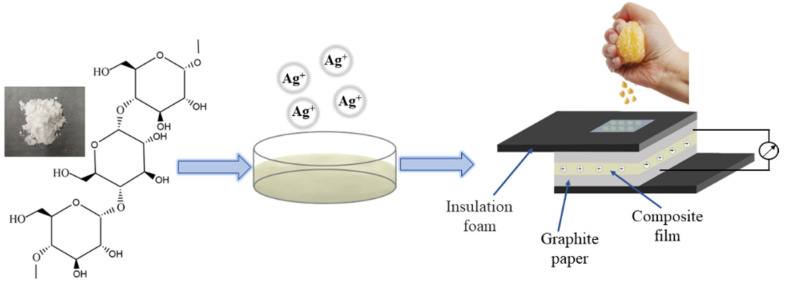
The synthesis route of ion-modified film and the flow chart of fruit juice detection.

Based on the above problems, we have prepared a composite functional film with a starch solution as the matrix, glycerol as the plasticizer and AgNO_3_ solution as the additive; this film responds sensitively to the pH value of fruit juice with the fast feedback of electrical signals (Figure 1). The mechanical properties, stability, hydrophilicity and pH response index of the composite films are analyzed and measured. Based on the above modern analytical techniques, we compare the effects of corn starch (CS) and amylopectin (CA) on the composite films and discuss the trends of the effects of ionic addition on the functionality of the composite films. The composite films respond sensitively to the pH value of the juice. This study provides a new method for the rapid, sensitive and nondestructive monitoring of the quality of liquid food, which has great application prospects in the field of rapid, nondestructive, and real-time digital monitoring of food quality.

## 2. Results

### 2.1. Mechanical Properties of Different Composite Films

**Figure 2:**
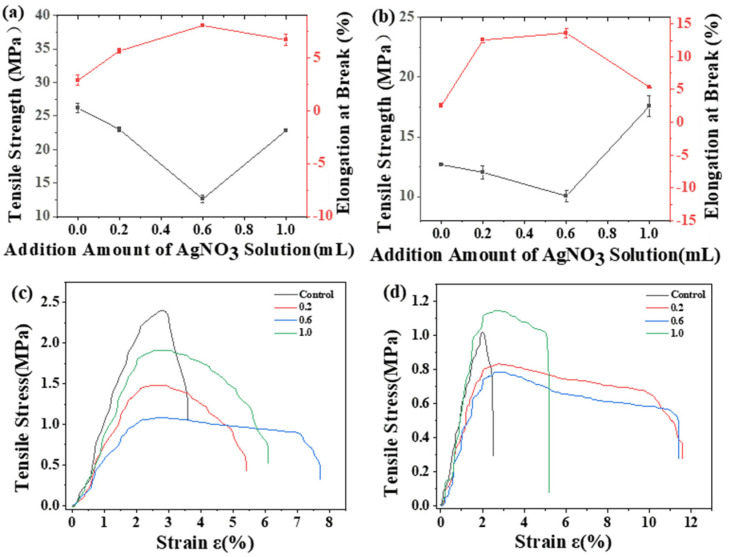
Tensile strength and the elongation at break of (**a**) CS/AgNO_3_ composite films and (**b**) CA/AgNO_3_ composite films; stress–strain curve of (**c**) CS/AgNO_3_ composite films, and (**d**) CA/AgNO_3_ composite films.

As shown in Figure 2a–d, the tensile strengths of the CS films were higher than those of the CA starch films, and the yield strengths of the CS films were higher than those of the CA starch films; this finding indicated that the service limits of the CS films were better than those of the CA films. The tensile strength of the two kinds of films showed trends of decreasing and then increasing with the increasing amount of added AgNO_3_. The elongation at break curves showed different degrees of increasing, with CS films showing less elongation at break than CA films. At 0.6 mL of AgNO_3_, both films showed large elongations at break. At this point, the tensile strength was the lowest, indicating that the film material was relatively soft. The tensile strength increased with increasing amounts of added AgNO_3_. According to an analysis, the high doping amount of silver nitrate solution enhanced the formation of hydrogen bonds and interactions in the starch molecules, resulting in enhanced rigidity properties of the CA composite films [26].

### 2.2. Morphological Characterization

**Figure 3:**
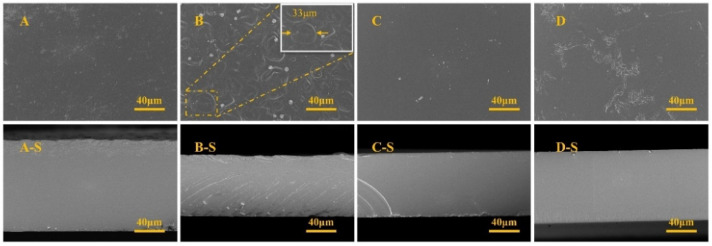
T SEM image of different films. (**A**): pure CS film, (**B**): CS/AgNO_3_-1.0 mL composite film, (**C**): pure CA film and (**D**): CA/AgNO_3_-1.0 mL composite film. X represents the surface topography of ABCD and X-S means the cross-section morphology.

To observe the changes occurring inside the films, the films were analyzed by electron microscopy. Scanning electron microscopy (SEM) images of the four starch films are shown in Figure 3. As shown in Figure 3, the addition of AgNO_3_ affected the starch surface finish to varying degrees and caused it to become rough. The surface of the CS films showed many irregular circular traces, with diameters of approximately 33 µm. The addition of AgNO_3_ resulted in a banded surface of the CA film. An analysis of the surface morphology showed that the addition of AgNO_3_ caused more changes to the CS. To characterize the structure of the film more clearly, the cross-section of the film was quenched at a low temperature, and its morphology was observed. As seen from Figure 3-X-S, the smoothness of the starch films without AgNO_3_ modification was better. With the addition of AgNO_3,_ the cross-section of the CS films showed signs of toughness fractures. However, the CA films did not change significantly and showed smoother morphological profiles. The surface morphology showed that the surface of the starch film was uneven and rough due to the addition of silver nitrate solution. The Ag^+^ in the silver nitrate solution potentially induced the agglomeration of the starch molecular chains, resulting in a decrease in the homogeneity of the starch emulsion; this phenomenon could cause floating on the surface of the film during the film formation process due to the density, thus leading to a rough surface. A similar phenomenon was reported previously [27].

### 2.3. XRD Structure Analysis

**Figure 4:**
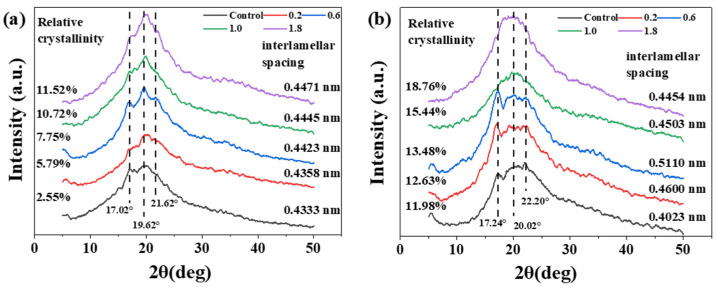
XRD curves of (**a**) CS/AgNO_3_ composite films and (**b**) CA/AgNO_3_ composite films.

To understand the crystallinity of the starch, X-ray diffraction (XRD) analysis of the starch was conducted, as shown in Figure 4. Figure 4 shows that the starch film material exhibited crystalline peaks at 17.0°, 19.6° and 21.6°. The addition of AgNO_3_ changed the relative crystallinity and layer spacing of the starch films. Figure 4a shows that AgNO_3_ increased the relative crystallinity of CS films and increased the layer spacing. The relative crystallinity reached a maximum of 11.52% when the doping amount of AgNO_3_ was 1.8 mL; additionally, the layer spacing reached a maximum at this time. With the increase in AgNO_3_, the crystallinity peak of the CS/AgNO_3_ composite film at 19.6° gradually became obvious. According to Figure 4b, the addition of AgNO_3_ solution enhanced the relative crystallinity of CA composite films, and the relative crystallinity increased with the addition increasing amounts. AgNO_3_ increased the layer spacing of CA films and reached a maximum at 0.5110 nm with an addition amount of 0.6 mL.

The analysis showed that the recrystallization of starch occurred, and the starch chains changed from disordered to ordered. During starch gelatinization, the ordered starch became disordered under influence of the actions of water and temperature, and the crystalline region opened completely. In starch film preparation, as the temperature decreased, the disordered starch chains in the high-energy state gradually tended to become ordered in the low-energy state due to the action of molecular potential energy, producing the phenomenon of gelation. When the moisture content of the starch film reduced to a certain level, the overall movement of the starch molecular chains substantially reduced or even froze, and the system was in a thermodynamic nonequilibrium state. As a result, the hydrogen bonds within or between the molecular chains rearranged, and the starch recrystallized. During recrystallization, amylose in starch formed a double helical structure; simultaneously, a double helical enriched region formed, which was eventually connected by intermolecular hydrogen bonds. In contrast, the recrystallization of CA occurred between the branches, with more crystalline regions forming within the molecule. Because amylopectin has numerous branched chains, the crystallinity of amylopectin formation exceeded that of the CS containing amylose. Therefore, the crystallinity of the CS film shown in Figure 4 was smaller than that of amylopectin. These findings were in agreement with the above analysis. Additionally, the crystallinity of the Ag^+^ ions was higher than that of the original starch, and the layer spacing increased to varying degrees. The analysis showed that Ag^+^ ions were enriched around the hydroxyl groups of the starch chains due to polarity, guiding the hydroxyl groups of other chain segments during recrystallization to a certain extent; this phenomenon would increase the proportion of recrystallization and cause an increase in crystallinity after the addition of Ag^+^ ions. Furthermore, due to the involvement of Ag^+^ ions in recrystallization, there were other substances in the crystal layer; thus, the layer spacing of the starch crystallization zone increased. The CA film had many crystalline branches within the molecular chains, resulting in weaker intermolecular linkages and lower tensile strength. The CS film was composed of amylose, which presented a helical structure between molecular chains, resulting in interchain bonding and crystallization under the guidance of ions; this film had a higher tensile strength than the CA film. The crystallization characteristics of the two films explained why the mechanical strength of the CS film was higher than that of the CA film in Figure 2.

### 2.4. Stability Analysis

**Figure 5:**
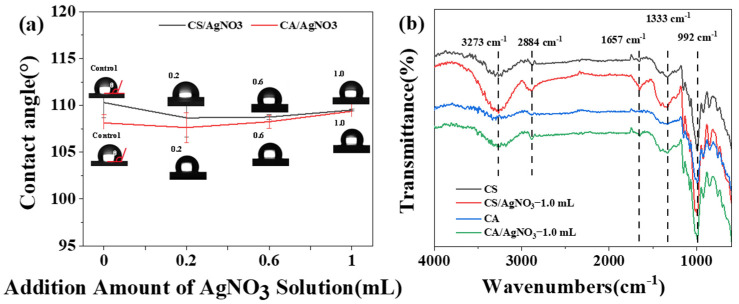
(**a**) Water contact angle of starch/AgNO_3_ composite films, (**b**) FTIR spectra of pure starch films and starch/AgNO_3_-1.0 mL composite films.

Figure 5a shows the water contact angle of the composite film in air. The water contact angle of the pure CS film was 110.3° and that of the pure CA film was 108.1°. As shown in Figure 5a, with the increase in the addition of AgNO_3_, the water contact angle for both starch-based composite films showed a trend of first decreasing and then increasing, reaching the lowest value at the addition of the amount of 0.2 mL. The reason for this phenomenon could be that silver ions were heavily doped at the starch chain segment points, and that the hydrophilic characteristics of ions led to a decrease in the water contact angle of the composite film. When the addition amount exceeded 0.2 mL, the ions aggregated to form a chelated starch gel shell; this aggregation made the starch film more hydrophobic, increasing the water contact angle. The water contact angle of the CS composite films was greater than that of the CA composite films. The higher the content of amylopectin in the film was, the better the hydrophilicity of the composite film and the lower the water contact angle [28].

### 2.5. Chemical Structure Analysis

Fourier transform infrared (FTIR) analysis was performed to observe the changes in the ion-modified starch groups. As shown in Figure 5b, starch/AgNO_3_ composite films showed no significant differences in terms of basic peak shapes from those of pure starch films. The -OH vibration absorption peak was at 3270 cm^−1^ [29], the -CH stretching vibration peak was near 2884 cm^−1^, and the C=O absorption peak was at 1657 cm^−1^. The distorted vibrational absorption peak of -OH appeared at 1333 cm^−1^ [30]. The stretching vibration absorption peak of C-O appeared at 992 cm^−1^, and the vibration absorption peak of the C-C skeleton appeared at 850 cm^−1^ [31]. As observed in Figure 5b, the addition of AgNO_3_ solution enhanced the -OH peak intensity but did not change the functional group type of the starch film. Compared to the pure starch film, the -OH stretching vibration peak at 3287 cm^−1^ was blueshifted. The smaller the wavelength number of the absorption peak was, the more intense the hydrogen bonding interactions were in the material; this finding further indicated that the addition of the silver nitrate solution enhanced the hydrogen bonding interactions [32].

**Figure 6:**
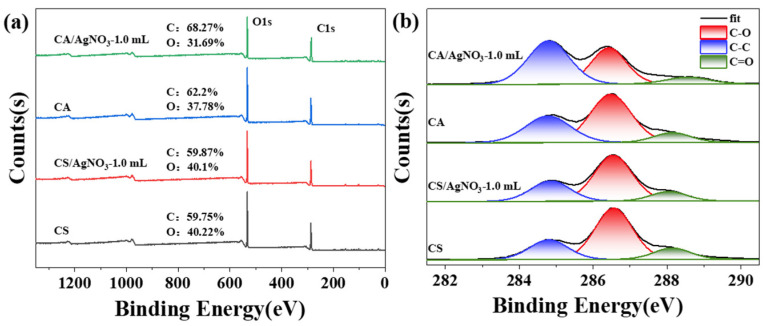
(**a**) XPS survey spectra, and (**b**) high-resolution C1s spectra of composite films.

The changes in groups and elements were not well observed by FTIR analysis; therefore, the films were analyzed by X-ray photoelectron spectroscopy (XPS) (Figure 6 and Table 1). The total XPS spectra of the four samples are shown in Figure 6a,b shows the C1s XPS spectra at high resolution. All samples exhibited strong peaks at 533 eV and 287 eV, corresponding to the binding energies of O1s and C1s, respectively. As shown in Table 1, the resolved C1s XPS spectra showed peaks at 284.8, 286.5 and 288.14 eV (Figure 6b), indicating the presence of C-C, C-O and C=O, respectively [33]. Table 1 suggests that the pure CS film and the CS/AgNO_3_ composite film had similar percentages of C and O elements; for amylopectin, the proportion of C in the CA/AgNO_3_ composite film was greater than that in the pure amylopectin film. As shown in Table 1, after adding silver nitrate solution, the proportion of C-C bonds in the composite films increased, and the corresponding proportions of C-O bonds and C=O bonds all decreased. Thus, the NO_3^−^_ ion had a weak oxidation ability in solution, and the starch film was oxidized and deoxidized during preparation; this phenomenon could reduce the proportion of oxygen [34]. In particular, the C=O bond in the CA/AgNO_3_ composite films only accounted for 4.62% of C1s. The doping of the silver nitrate solution did not change the structure of the functional groups, but the percentage of different bonds in the structure changed significantly.

### 2.6. Chemical Structure Analysis

**Figure 7:**
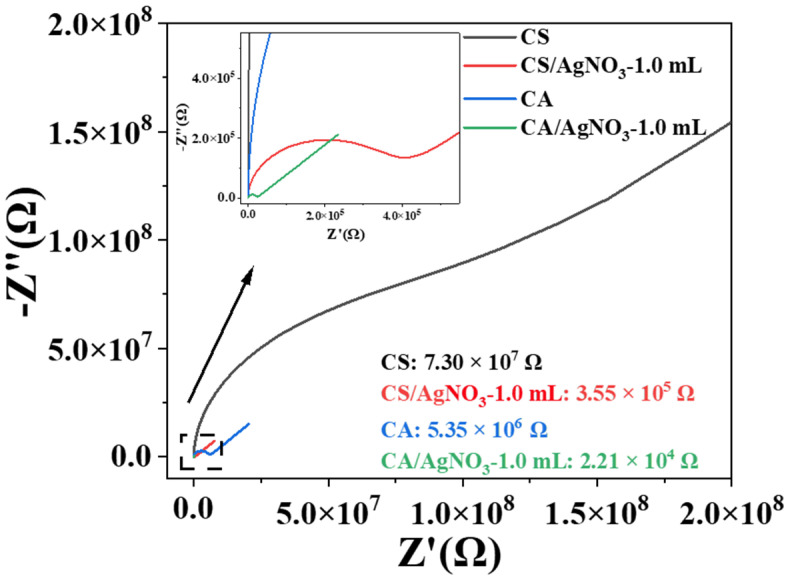
EIS of pure starch films and starch/AgNO_3_-1.0 mL composite films.

Figure 7 shows the electrochemical impedance spectroscopy (EIS) of the pure starch film and the starch/AgNO_3_-1.0 mL composite films. The EIS diagram features a semicircle in the high-frequency region and a straight line in the medium-to-low-frequency regions (Figure 7). The semicircular arc in the high-frequency region is the charge transfer impedance (Rc) caused by the gain and loss of electrons, while the straight line in the medium and low frequency regions is related to the solid diffusion process. The smaller the diameter of the semicircular arc of the impedance curve is, the faster the charge transfer rate and the lower the electron transfer resistance will be [35]. According to the EIS, after circuit fitting, the doping of the AgNO_3_ solution reduces the resistance of the composite films and enhances their electrical conductivity. The addition of the AgNO_3_ solution enhances the electrical conductivity of the film, probably due to the formation of ionic hydrates in the composite film, decreasing the resistance value of the composite film. By comparing the CS and CA films, the resistance value of the CS film is determined to be greater than that of the CA film under the same test conditions. According to the EIS and XRD analyses, this phenomenon may be caused by the amylose in the CS film, which is associated with the linking of the starch molecule between the chains, making the film-forming structure tighter and resulting in its poor electrical conductivity.

To show the conductivity gap between the films more clearly, the current strengths of the films in different pH solutions were measured. The modified films had sensitive pH responses to the changes in current.

The currents in the different environments were measured by adding drops of acid–base solutions of diverse pH values to the self-assembled parts of the film, changing the acid–base environment existing in the film. The different pH solutions caused a change in the current of the film, thus characterizing the acidity of the solution by the current.

**Figure 8:**
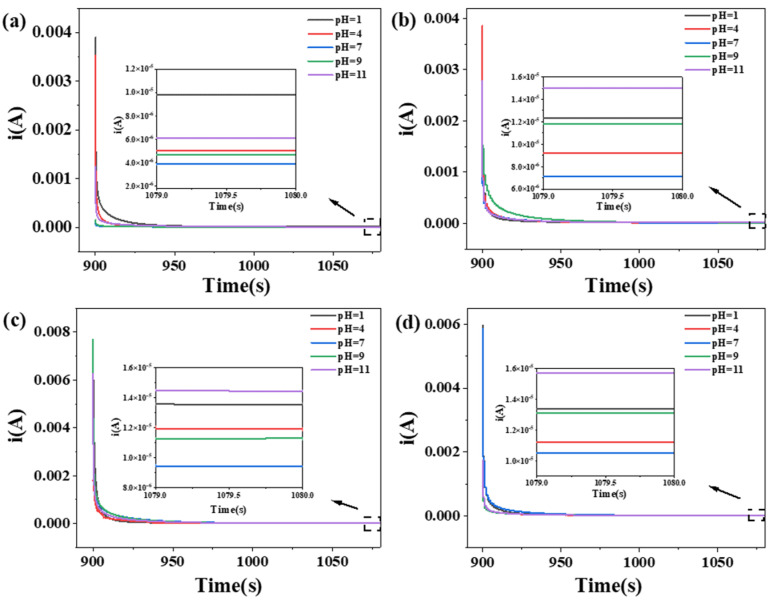
Steady current at different pH value of (**a**) pure CS film, (**b**) CS/AgNO_3_-1.0 mL composite film, (**c**) pure CA film and (**d**) CA/AgNO_3_-1.0 mL composite film.

As shown in Figure 8, the conductivity of the starch/AgNO_3_ composite films was greater than that of the pure starch films, which could have been caused by the ions being embedded in the films. The addition of the AgNO_3_ solution formed hydrated ions, which in turn enhanced the conductivity of the films, resulting in higher steady currents than those of the pure starch films. Under different pH conditions, the smallest stable current values were obtained at pH = 7. The more acidic or alkaline the solution was, the more the stable current values increased, making the current conducted by the film positively correlated with the ion concentration. According to the CA test results, the embedding of the AgNO_3_ solution effectively reduced the resistance of the starch film, thereby enhancing its conductivity. This was consistent with the EIS results.

The stable currents for CS films ranged from 3.92 × 10^−6^ A to 9.76 × 10^−6^ A at different pH values; for CS/AgNO_3_-1.0 mL composite films, the currents ranged from 9.41 × 10^−6^ A to approximately 1.44 × 10^−5^ A. The stable currents for pure CA films ranged from 6.92 × 10^−6^ A to 1.48 × 10^−5^ A at different pH values; for CA/AgNO_3_-1.0 mL composite films, the currents ranged from 1.01 × 10^−5^ A to approximately 1.54 × 10^−5^ A. 

To verify the pH response of the modified starch films, we used orange juice after 14 days of storage as the test liquid. The current was measured by adding drops of orange juice to a device prepared from modified starch.

**Figure 9:**
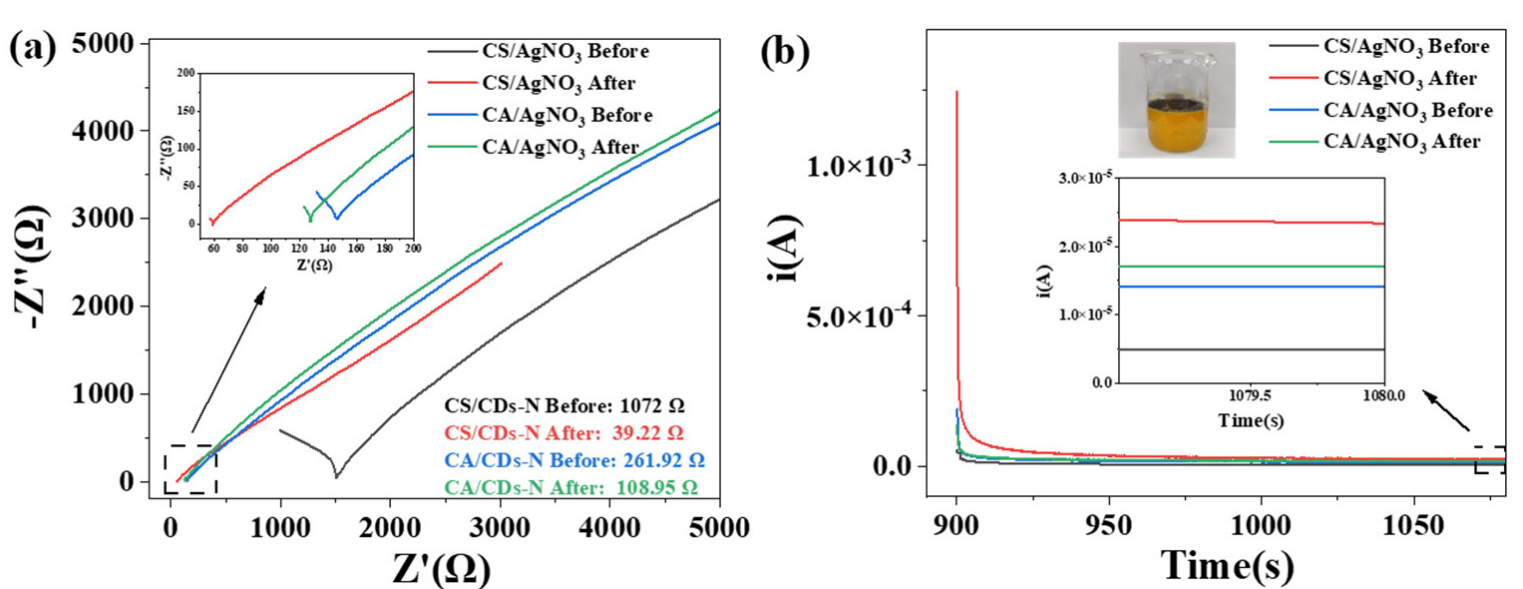
EIS (**a**) and Steady current (**b**) of starch/AgNO_3_-1.0 mL composite films.

The pH of fresh orange juice was approximately 3.38, and the pH of the spoiled orange juice was 2.68, indicating that it somewhat decreased. As shown in Figure 9, before deterioration, orange juice was added to the CS/AgNO_3_-1.0 mL composite film and CA/AgNO_3_-1.0 mL composite film devices and stabilized for 30 min; then, it fully entered the film devices. The obtained stable current values were 4.42 × 10^−6^ A and 1.39 × 10^−5^ A for the CS/AgNO_3_-1.0 mL and CA/AgNO_3_-1.0 mL devices, respectively. After spoilage, the measured stable current values were 2.46 × 10^−5^ A and 1.76 × 10^−5^ A for the CS/AgNO_3_-1.0 mL and CA/AgNO_3_-1.0 mL devices, respectively. Comparing the stable current values of the two composite films shows that the difference in current between the CS/AgNO_3_-1.0 mL composite films before and after the spoilage of the orange juice was obvious, while the difference in current between the CA/AgNO_3_-1.0 mL composite film was neither obvious nor significant. As seen from Figure 9a, the resistance of the CS/AgNO_3_ composite film was 1072 Ω before juice spoilage and 39.22 Ω after spoilage, with a difference of nearly 1030 Ω; the difference for the CA/AgNO_3_ composite film was only 153 Ω. The CS/AgNO_3_ composite film was more beneficial for detecting juice spoilage. Information on the spoilage of orange juice was obtained from both the current and impedance values of the modified film device, providing new ideas for the real-time monitoring of food safety.

## 3. Discussion

In conclusion, starch/AgNO_3_ composite films were prepared by a simple solution casting method. The tensile strength of the CS/AgNO_3_-1.0 composite film reached 22.82 MPa, and the elongation at break was 6.7%; the tensile strength of the CA/AgNO_3_-1.0 composite film reached a maximum of 17.58 MPa, and its elongation at break was 5.35%. The addition of AgNO_3_ increased the crystallinity of the starch films to varying degrees, with the crystallinity of CA increasing more than that of CS; the highest crystallinity was 18.76% for CA. Due to the high crystallinity of the recrystallized CA films, the stability was superior. The addition of ions made the starch films more hydrophilic and provided a basis for the films to respond to the pH of the liquid. The addition of ions resulted in a higher C-C bond ratio, making the pH-responsive device more stable. At an addition level of 1.0 mL, the composite film had good conductivity, making it sensitive to different pH solutions. The CS/AgNO_3_ composite film was tested on orange juice and found to have more sensitive resistance and current response, which was more conducive for application in the quality monitoring of fruit juices. Overall, the composite films prepared by the authors showed certain application prospects in the field of food and biodegradable films, providing new strategies for juice detection and digital monitoring.

## 4. Materials and Methods

### 4.1. Materials

Corn starch (CS) and corn amylopectin (CA) were purchased from Shanghai Aladdin Biochemical Technology Co., Ltd. (Shanghai, China), AR grade. Silver nitrate (AgNO_3_) was purchased from Guangdong Xilong Chemical Co., Ltd. (Shantou, China), AR grade. All solution were prepared with ultrapure water.

### 4.2. Methods

The component interaction of film samples was studied with a Fourier transform infrared spectrometer (FTIR) (Waltham, MA, USA) with a resolution of 4 cm^−1^. The elemental composition of the films was observed using Thermo Scientific K-Alpha X-ray photoelectron spectrometer (XPS) (Thermo Fisher Scientific, Shanghai, China). The X-ray diffractometer (XRD), model max220 (Neo-D, Japan), with Cu Kα radiation (λ = 1.5406 nm) and the relative crystallinity was analyzed using Jade 6 software (International Centre for Diffraction Data, Newtown, PA, USA). The morphology and microstructure of the films were observed by scanning electron microscopy (SEM). The mechanical properties of the film, namely tensile strength and elongation at break, were measured by XLM (PC) with intelligent electronic tensile testing machine. The hydrophilicity and hydrophobicity of the film samples were tested by a JC2000D3R (Shanghai Zhongchen Digital Technology Equipment Co., Ltd., Shanghai, China) water contact angle measuring instrument. The impedance of the dried film samples was measured by CHI760E electrochemical workstation (Shanghai Chenhua Instrument, Shanghai, China). The test frequency was 0.1 Hz~1 MHz, and the amplitude was 0.01 V. Layer-by-layer self-assembled devices were produced for electrochemical testing. The device was prepared by coating graphite paper and the innermost film sample with insulating collodion. The liquid to be measured was dropped on the surface of the film, and the steady current and resistance were determined using chronoamperometry and electrochemical impedance spectroscopy.

### 4.3. Film Preparation

The starch/AgNO_3_ composite films were prepared by simple solution casting. The starch solution was prepared by dissolving starch (4 g) in 96 mL of ultrapure water with the aid of stirring and adding 1.4 g of glycerol. The solution was transferred to a 90 °C water bath and stirring was continued until the solution was completely gelatinized. Then, the different levels of AgNO_3_ solution (10 mmol/L) were added to the starch solution and stirring was continued for another 10 min. The homogeneous film solution was poured into the Petri dish (d = 15 cm) and then baked in a constant temperature oven at 45 °C for 6 h. The composite film peeled off from the Petri dish was coded as starch/AgNO_3_-x, where x indicates the amount of AgNO_3_ solution added to the starch film. For example, CS/AgNO_3_-1.0 means that the amount of AgNO_3_ solution doped in the CS film is 1.0 mL.

## Figures and Tables

**Table 1:** Element proportion and chemical bond for different composite films.

	CS	CS/AgNO_3_-1.0	CA	CA/AgNO_3_-1.0
C1s	59.75%	59.87%	62.20%	68.27%
O1s	40.22%	40.1%	37.78%	31.69%
C-C	24.84%	27.88%	34.08%	37.63%
C-O	62.11%	60.61%	55.87%	53.76%
C=O	13.04%	11.52%	10.06%	4.62%

## Data Availability

The data presented in this study are available on request from the corresponding author.

## References

[1] Serge S., Stephen W., Sonia A., Conny J., Jody H., Lise K., Reimund P., Goedele V.B. (2022). Revisiting food security in 2021: An overview of the past year. Food Secur..

[2] Jin T., Zhong T.Y. (2022). Changing rice cropping patterns and their impact on food security in southern China. Food Secur..

[3] He C.Y., Liu Z.F., Xu M., Ma Q., Dou Y.Y. (2017). Urban expansion brought stress to food security in China: Evidence from decreased cropland net primary productivity. Sci. Total Environ..

[4] Seto K.C., Ramankutty N. (2016). Hidden linkages between urbanization and food systems. Science.

[5] Liu S., Hou M.Y. (2022). Spatiotemporal differences, dynamic evolution and trend of the coupled coordination relationship between urbanization and food security in China. Foods.

[6] Xiang M.T., Li Y., Yang J.Y., Lei K.G., Li Y. (2021). Heavy metal contamination risk assessment and correlation analysis of heavy metal contents in soil and crops. Environ. Pollut..

[7] Tang J., Zhang Q., Zhou J., Fang H.C., Yang H.F., Wang F. (2021). Investigation of pesticide residue removal effect of gelatinized starch using surface-enhanced Raman scattering mapping. Food Chem..

[8] Yesim O., Nariman E.A., Fatih O. (2022). Antimicrobial effect of laurel essential oil nano-emulsion on food-borne pathogens and fish spoilage bacteria. Food Chem..

[9] Wang L., Huang X.Y., Wang C.Q., Tian X.Y., Chang X.H., Ren Y., Yu S.S. (2021). Applications of surface functionalized Fe_3_O_4_ NPs-based detection methods on food safety. Food Chem..

[10] Xu J., Cao Z., Zhang Y.L., Yuan Z.L., Lou Z.M., Xu X.H., Wang X.K. (2018). A review of functionalized carbon nanotubes and graphene for heavy metal adsorption from water: Preparation, application, and mechanism. Chemosphere.

[11] Martins G.C., Coutinho T.E., Silva T.L., Andreani T., Silva A.M. (2022). Neurotoxicity assessment of four different pesticides using in vitro enzymatic inhibition assays. Toxics.

[12] Ibarra B.L.M.E., Hernandez S.R., Kergaravat S.V. (2021). Glyphosate detection from commercial formulations: Comparison of screening analytic methods based on enzymatic inhibition. Int. J. Environ. Anal. Chem..

[13] Patil D.Y., Khadke N.B., Patil A.A., Borhade A.V. (2022). Amino-quinoline based colorimetric chemosensor for Cu^2+^ detection. J. Anal. Chem..

[14] Wang W.Z., You Y.S., Gunasekaran S. (2021). LSPR-based colorimetric biosensing for food quality and safety. Compr. Rev. Food Sci. Food Saf..

[15] Jin Q., Feng L., Wang D.D., Wu J.J., Hou J., Dai Z.R., Sun S.G., Wang J.Y., Ge G.B., Cui J.N. (2016). A highly selective near-infrared fluorescent probe for carboxylesterase 2 and its bioimaging applications in living cells and animals. Biosens. Bioelectron..

[16] Wafula E.N., Onduso M., Wainaina I.N., Buve C., Kinyanjui P.K. (2022). Antinutrient to mineral molar ratios of raw common beans and their rapid prediction using near-infrared spectroscopy. Food Chem..

[17] Simona D., Volha S., Valeria T., Achim K., Dana B., Martin S., Ivana M., Boris Z. (2021). Assessment of biotechnologically important filamentous fungal biomass by Fourier transform Raman spectroscopy. Int. J. Mol. Sci..

[18] Lima T.K., Musso M., Bertoldo M.D. (2020). Using Raman spectroscopy and an exponential equation approach to detect adulteration of olive oil with rapeseed and corn oil. Food Chem..

[19] Orzel J., Swit P. (2021). Comparison of quantitative detection methods based on molecular fluorescence spectroscopy and chromatographic techniques used for the determination of bisphenol compounds. Int. J. Mol. Sci..

[20] Wang S.M., Yang C.Y., Liu Y.Q., Wang Y.R., Zhao Q. (2022). Determination of heterocyclic aromatic amines in various fried food by HPLC-MS/MS based on magnetic cation-exchange resins. Food Anal. Method..

[21] Zhang X.Q., Chen C.Y., Peng D.P., Zhou Y.Z., Zhuang J.L., Zhang X.J., Lei B.F., Liu Y.L., Hu C.F. (2020). pH-responsive carbon dots with red emission for real-time and visual detection of amines. J. Mater. Chem. C.

[22] Hu Z., Wang H.L., Li L.L., Wang Q., Jiang S.W., Chen M.M., Li X.J., Jiang S.T. (2021). pH-responsive antibacterial film based polyvinyl alcohol/poly (acrylic acid) incorporated with aminoethyl-phloretin and application to pork preservation. Food Res. Int..

[23] Deng P.H., Xu Z.F., Kuang Y.F. (2013). Electrochemically reduced graphene oxide modified acetylene black paste electrode for the sensitive determination of bisphenol A. J. Electroanal. Chem..

[24] Hao W.X., Ge Y., Qu M.R., Wen Y.P., Liang H. (2022). A simple rapid portable immunoassay of trace zearalenone in feed ingredients and agricultural food. J. Food Compos. Anal..

[25] Kundan S., Kenneth S., Joseph A.K., Ailing T., Yong Z., Gregory L.R., Alan X.W. (2019). Biological photonic crystal-enhanced plasmonic mesocapsules: Approaching single-molecule optofluidic-SERS sensing. Adv. Opt. Mater..

[26] Kim H.Y., Jane J.L., Lamsal B. (2017). Hydroxypropylation improves film properties of high amylose corn starch. Ind. Crops Prod..

[27] Zuo Y., Gu J., Yang L., Yang L., Qiao Z.B., Tan H.Y., Zhang Y.H. (2013). Synthesis and characterization of maleic anhydride esterified corn starch by the dry method. Int. J. Biol. Macromol..

[28] Li C., Gong B. (2020). Insights into chain-length distributions of amylopectin and amylose molecules on the gelatinization property of rice starches. Int. J. Biol. Macromol..

[29] Maria J.B., Valeria C.B., Delia E.L., Maria A.G. (2015). Chitosan molecular weight effect on starch-composite film properties. Food Hydrocolloids..

[30] Ren L.L., Yan X.X., Zhou J., Tong J. (2017). Influence of chitosan concentration on mechanical and barrier properties of core starch/chitosan films. Int. J. Biol. Macromol..

[31] Tedeschi A.M., Caprio F.D., Piozzi A., Pagnanelli F., Francolini I. (2022). Sustainable bioactive packaging based on thermoplastic starch and microalgae. Int. J. Mol. Sci..

[32] Liu D., Bian Q.B., Li Y., Wang Y.R., Xiang A.M., Tian H.F. (2016). Effect of oxidation degrees of graphene oxide on the structure and properties of poly(vinyl alcohol) composite films. Compos. Sci. Technol..

[33] Yu B.Y., Kwak S.Y. (2012). Carbon quantum dots embedded with mesoporous hematite nanospheres as efficient visible light-active photocatalysts. J. Mater. Chem. A.

[34] Luo Z.G., Zou J.F., Chen H.M., Cheng W.W., Fu X., Xiao Z.G. (2016). Synthesis and characterization of amylose-zinc inclusion complexes. Carbohydr. Polym..

[35] Xu T., Ding X.T., Shao C.X., Song L., Lin T.Y. (2018). Electric power generation through the direct interaction of pristine graphene-oxide with water molecules. Small.

